# Androgen receptor agonist and antagonist reduce response of cytokine‐induced killer cells on prostate cancer cells

**DOI:** 10.1111/jcmm.17923

**Published:** 2023-08-28

**Authors:** Thanakorn Pungsrinont, Margret Ann Schneider, Aria Baniahmad

**Affiliations:** ^1^ Institute of Human Genetics, Jena University Hospital Friedrich Schiller University Jena Germany

**Keywords:** androgen receptor, cancer cell senescence, cytotoxic lymphocytes, immune escape, immune killing assays, onco‐immunology, prostate cancer

## Abstract

Despite many advances, prostate cancer (PCa) is still the second most frequently diagnosed cancer and fifth leading cause of cancer death in men worldwide. So far, the promising field of onco‐immunology has not yet provided a satisfactory treatment option for PCa. Here we show that the ex vivo expansion and activation of cytokine‐induced killer (CIK) cells isolated from primary peripheral blood mononuclear cells induce immune‐mediated apoptosis in both human PCa LNCaP and C4‐2 cells. Interestingly, pretreating LNCaP and C4‐2 cells with either androgen or the androgen receptor (AR) antagonist enzalutamide mediates resistance to this immunogenic attack. This is associated with a reduction of both total cell loss and apoptosis levels suggesting one possible mechanism blunting onco‐immunological activity. The data also suggest that secreted factors from AR ligand‐treated PCa cell suppress lymphocyte proliferation. Further, we analysed immune‐mediated killing activity using conditioned media from LNCaP and C4‐2 treated cells. The obtained data suggest that the conditioned media from PCa treated cells does not influence a measurable lymphocyte‐mediated apoptosis. However, analysing clonal expansion of activated lymphocytes, the androgen‐derived conditioned media suppresses lymphocyte proliferation/expansion suggesting inhibition of onco‐immunological activity by pretreatment of PCa cells with AR ligands.

## 1 INTRODUCTION

Prostate cancer (PCa) is one the most diagnosed cancers and the second leading cause of cancer‐related death in men in Western countries.^1^ The androgen receptor (AR) is a key factor controlling tumorigenesis of PCa. AR is activated by androgens and can be inactivated by receptor antagonists such as the second‐generation antagonist enzalutamide (ENZ) used to treat castration‐resistant PCa (CRPC). In line with this, androgen deprivation therapy combined with AR antagonists is used most often for hormone therapy of PCa. Interestingly, an emerging treatment in ongoing Phase 2 clinical trials^2^ is the bipolar androgen therapy (BAT), which is based on the paradoxical inhibition of PCa cell growth by supraphysiological androgen levels (SAL).^3^, ^4^ BAT, which includes the cyclic treatment of patients with SAL and continuous treatment with androgen deprivation, is suggested to reduce vulnerable phases and the development of adaptive resistance mechanisms through the rapid switching between supraphysiological and castration‐level androgen levels. Interestingly, both ENZ and SAL induce cellular senescence in model systems of human PCa cells including cell lines representing castration sensitive and CRPC cells, PCa spheroids, and ex vivo‐treated human patient samples derived from prostatectomies^5^, ^6^ known as therapy‐induced cellular senescence.^7^ Especially metastasized CRPC still poses a great challenge, with a median survival of only approximately 42 months.^8^ So far, the only onco‐immunological treatment approved specifically for PCa was Sipuleucel‐T, which was, however, withdrawn from use in the European Union in 2015 due to a lack of quantifiable added benefits.^9^, ^10^ Since 2010, to our knowledge no other onco‐immunological treatment has been approved specifically for PCa. However, the use of immune checkpoint inhibitors as mono‐ or combined therapies, BiTE® (bispecific T‐cell engager) immune therapies or chimeric antigen receptors for metastasized CRPC is being studied in multiple Phase 2 and 3 clinical trials.^11^

The reason and mechanisms behind this lack of success are not yet fully understood. Some explanations include the fact that PCa seems to have a ‘cold’ or immunosuppressive tumour immune microenvironment,^12^ as well as the high tumour heterogeneity found in PCa. The high tumour heterogeneity may explain the surprisingly robust response to onco‐immunological treatments found in a small subgroup of patients compared to the complete lack or marginal response found in the majority of PCa patients.^13^

Here, we aimed to analyse the response of PCa cells pretreated with the AR antagonist ENZ or androgen at SAL to cytokine‐induced killer (CIK) cells. This study includes the analysis of senescence associated secretory phenotype (SASP) since both ENZ and SAL treatment induce cellular senescence in both castration‐sensitive and CRPC cells.^5^, ^6^, ^14^ The hypothesis was that these AR ligands may change the secretion of factors by PCa cells and thereby influence clonal expansion and cytotoxic activity of CIK cells.

For that purpose, primary peripheral blood mononuclear cells (PBMCs) were isolated from the population of whole blood samples. Maturation and differentiation of immune cells were induced. Followed by flow cytometric verification, the CD3^+^ CD8^+^ lymphocyte population was expanded into the dominant subgroup of the lymphocyte population and incubated with untreated and treated LNCaP cells (a model for castration‐sensitive PCa) or C4‐2 human PCa cell line (a cell model system for CRPC). In addition, conditioned media from AR ligand treated cells were used and analysed for suppression of lymphocyte proliferation/expansion, cytotoxic‐induced apoptosis, and cellular senescence.

## 2 RESULTS

### 2.1 Isolation and maturation of primary peripheral blood mononuclear cells (PBMCs) from healthy donors

**FIGURE 1:**
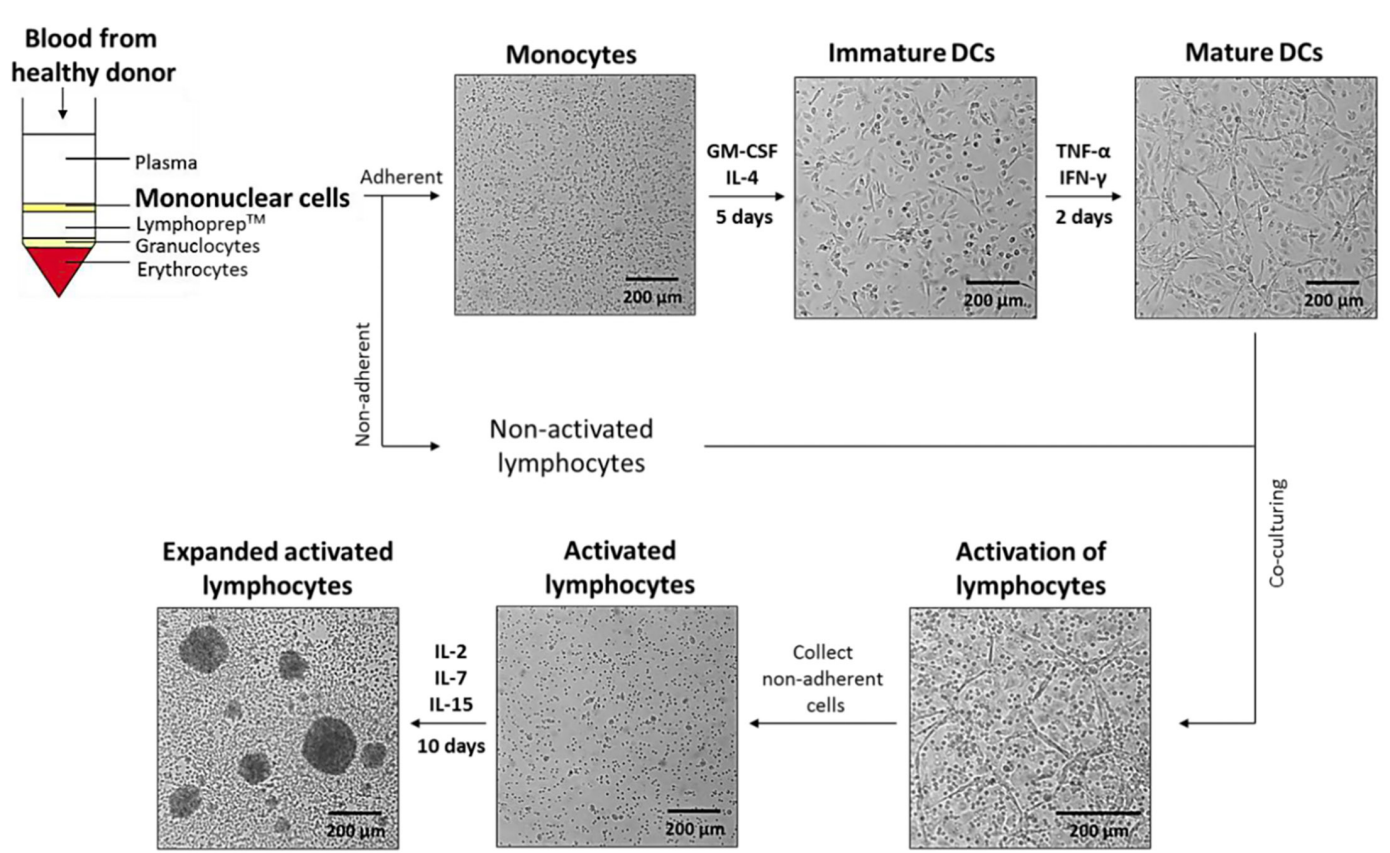
Schematic view for culturing and activation of human immune cells. Illustration of experimental setup for culturing and activation of immune cells adapted from Junking et al.^15^ Isolation of peripheral blood mononuclear cells (PBMCs) from healthy donors, culture conditions to generate mature dendritic cells (DC), lymphocyte activation, and the clonal expansion of lymphocytes.

**FIGURE 2:**
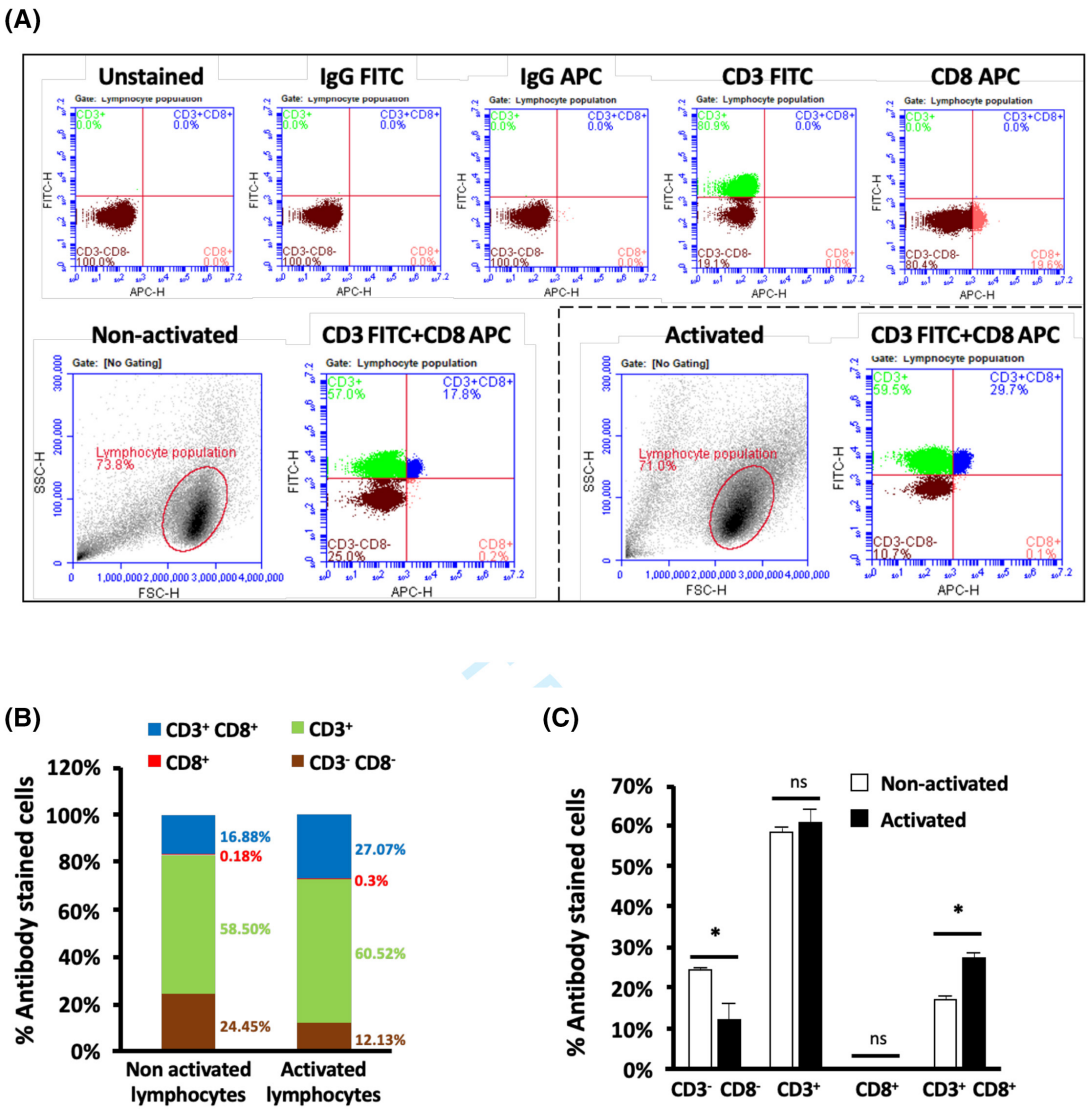
Activation of human immune cells. (A) Detection of CD3^+^ and CD8^+^ lymphocyte populations by flow cytometry. Detection intensity threshold was set according to unstained, IgG FITC, IgG APC, CD3 FITC, and CD8 APC‐stained populations to establish the quadrant plot gate. Green = CD3^+^; red = CD8^+^; blue = CD3^+^ CD8^+^; brown = CD3^−^ CD8^−^. (B) Percentage of CD3^+^ and CD8^+^ antibody‐stained cells analysed from flow cytometry data. Numbers indicates the mean percentage of four different populations within nonactivated or activated lymphocytes populations. The mean percentage was calculated from three independent experiments (*N* = 3). (C) Separated histogram data from (B) as mean + SEM. Statistical analysis was performed by using two‐tailed unpaired t‐test comparing activated population to nonactivated population. *p ≤ 0.05; ns, not significant.

PBMCs were isolated from whole blood samples from healthy donors (Figure 1). Mononuclear cells were isolated as monocytes and nonactivated lymphocytes, as adherent and nonadherent cells, respectively (Figure 1). By treating with GM‐CSF, IL‐4, TNF‐α, and IFN‐γ, the adherent monocytes were cultured and differentiated into mature dendritic cells (DC). The nonadherent, nonactivated lymphocytes cells were activated by co‐culturing with mature DC. The activated lymphocytes were further expanded by treatment with IL‐2, IL‐7 and IL‐15 for 10 days. The differentiation of antigen presenting cells or monocyte‐derived mature DC was visualized by morphological changes from a spherical to a dendritic shape. The activated lymphocytes were cultured and verified by colony formation (Figure 1). Successful cytokine induced activation and clonal expansion was confirmed by an increased population of CD3^+^ CD8^+^ killer cells within the lymphocyte population (Figure 2A) with quantifications (Figure 2B,C). The data indicate the differentiation of peripheral blood monocytes into mature DC and the increase in killer lymphocyte subpopulation.

### 2.2 AR ligand‐induced cellular senescent PCa cells are resistant to lymphocyte‐mediated apoptosis

**FIGURE 3:**
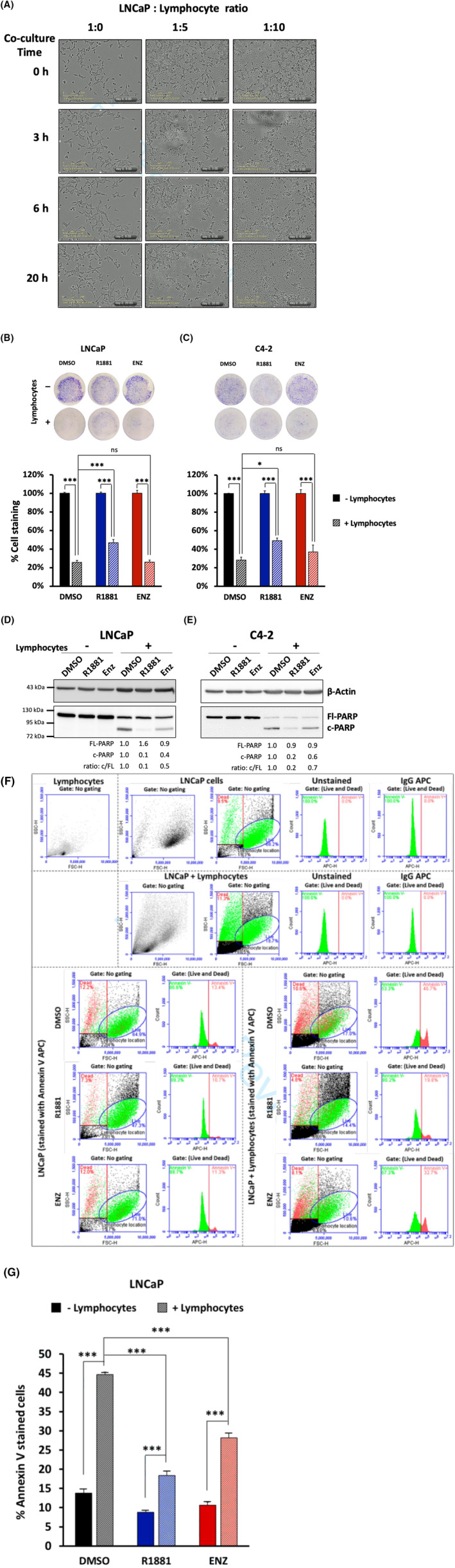
AR ligand‐induced cellular senescent PCa cells are resistant to lymphocyte‐mediated apoptosis. (A) LNCaP cells were killed by activated lymphocytes. LNCaP cells were co‐cultured with activated lymphocytes using different indicated ratios of activated lymphocytes to treat LNCaP cells. Pictures were captured from live imaging microscope at 0, 3 and 6 h after co‐culturing was started. The results suggest 1:5 ratio and 6 h to be the maximum co‐culturing ratio and incubation time for analysing the lymphocyte‐mediated cytotoxicity on LNCaP cells. (B, C) AR ligands change sensitivity to onco‐immunological response of LNCaP (B) and C4‐2 cells (C). Cells were treated for 72 h with 1 nM R1881, 1 μM ENZ or 0.1% DMSO as solvent control. Thereafter, PCa cells were co‐cultured with or without activated lymphocytes in a ratio of 1:5 (PCa cells: lymphocytes). Top panel: Representative pictures of crystal violet staining after co‐culture for 6 h. Lower panel: Percentage of cell survival calculated from crystal violet staining. Cell survival without lymphocyte co‐culture was set arbitrarily as 100%. Bar graphs are shown as mean + SEM from total of six technical replicates (*n* = 6) of three independent experiments. (D, E) Detection of the apoptotic marker cleaved PARP (c‐PARP) was performed by western blotting and normalized to β‐Actin for treated LNCaP (D) and C4‐2 cells (E). Upper and middle numbers indicate normalized FL‐PARP and c‐PARP band intensities relative to DMSO+lymphocytes. Lower numbers indicate the ratios of c‐PARP versus FL‐PARP levels. (F) Detection of the apoptotic marker Annexin V was performed by flow cytometry after co‐cultured for 3 h. LNCaP and lymphocytes populations were first identified for correct gating. Detection intensity threshold was set according to unstained and IgG APC‐stained populations (green = Annexin V negative; red = Annexin V positive). (G) Percentage of Annexin V stained LNCaP cells analysed from the data of flow cytometry. Bar graphs are shown as mean + SEM from six technical replicates (*n* = 6) of two independent experiments. Statistical analyses in (B), (C), and (G) were performed by using one‐way ANOVA followed by Bonferroni's multiple comparisons test. ***p < 0.001; *p < 0.05; ns, not significant.

Lymphocyte‐mediated killing assays of LNCaP cells were performed using activated lymphocytes. In pretests the co‐culture incubation time as well as the ratio between PCa cells and lymphocytes were analysed (Figure 3A). The results suggest a 1:5 ratio to be the maximum co‐culture cell ratio and the duration of 6 h is a suitable incubation time to analyse the lymphocyte‐mediated cytotoxic effect on LNCaP cells.

Previous study show that either AR agonist at SAL or AR antagonist induce cellular senescence in PCa cells.^5^, ^6^, ^14^ In line with this, AR ligand‐treated PCa cells seem to be apoptotic resistant. Thus, it was analysed whether the AR‐agonist or AR antagonist treated cells show a different response to lymphocytes. For that purpose, lymphocyte‐mediated killing assays of PCa cells were performed by co‐culturing the androgen‐sensitive LNCaP or the CRPC C4‐2 cells with activated lymphocytes. Note that, the AR activity in CRPC including C4‐2 cells is higher without androgen treatment compared to LNCaP cells suggesting that the AR can act ligand‐independently.^16^ This is in line with observations that using concentration series, the basal level of AR target gene expression is higher and the fold inducibility by androgen is lower in CRPC cells compared to LNCaP cells.^5^ Nevertheless, either ENZ or SAL can induce cellular senescence in CRPC, which was suggested as one underlying molecular mechanism to inhibit tumour growth.^6^, ^14^ In this experiment, LNCaP and C4‐2 cells were treated 72 h prior to addition of activated lymphocytes with the AR agonist methyltrienolone (R1881) at SAL or the AR antagonist ENZ. DMSO served as solvent control. In contrast to dihydrotestosterone, which is rapidly metabolized and its metabolites may act as oestrogen receptor beta agonists,^17^ the much less metabolisable synthetic androgen R1881 and thus more AR‐specific androgen was used. Moreover, a dose response of both ENZ and R1881‐treated cells to induce cellular senescence by these AR ligands is indicated in supplemental Figure S1 revealing a similar level of induction of cellular senescence by employing 1 nM R1881 and 1 μM ENZ.^5^, ^6^ Therefore, these concentrations were used throughout. In agreement with these data, AR antagonists are in general used at much higher concentrations for bioactivity compared to androgens. One underlying reason is the lower affinity of AR antagonists compared to AR agonists.

A resistance towards apoptosis is likely to result in better survival. Therefore, to analyse relative change of PCa cells after co‐culturing with lymphocytes, crystal violet staining were performed after co‐culture for 6 h (Figure 3B,C). The data suggest that cell survival of both LNCaP and C4‐2 cell lines was reduced after lymphocyte co‐culture. This reflects that LNCaP and C4‐2 cells were killed by lymphocytes. The quantification of crystal violet staining confirms a potent lymphocyte‐mediated killing of both LNCaP and C4‐2 cell lines (Figure 3B,C). Interestingly, the data also suggest that androgen‐treated PCa cells seem to be resistant to lymphocyte‐mediated killing (Figure 3B,C).

For confirmation, the apoptosis marker c‐PARP was used in Western blotting experiments. While in the absence of lymphocytes no c‐PARP was detectable, treating with co‐culturing activated lymphocytes for 1.5 h, c‐PARP was detected most prominently in the solvent control lane of extracts from both LNCaP and C4‐2 cells (Figure 3D, E). This confirms earlier data that the AR ligands themselves do not induce apoptosis rather cellular senescence.^6^, ^14^ LNCaP or C4‐2 cells co‐cultured with lymphocytes showed increased apoptotic marker c‐PARP levels indicating successful immune‐cell mediated killing of PCa cells. Interestingly, treating cells with R1881 or ENZ indicated less presence of the apoptotic c‐PARP compared to the DMSO‐solvent control (Figure 3D,E). Thus, the data suggest that AR agonist‐ or antagonist‐treated PCa cells are resistant to lymphocyte‐mediated apoptosis with agonist exhibiting a stronger resistance compared to ENZ treatment.

To further validate these findings, flow cytometry was used for detection of Annexin V as another apoptotic marker. The LNCaP and lymphocyte population as control were first analysed for appropriate gating (Figure 3F). The threshold based on detection intensity was set relative to unstained or IgG APC‐stained population (red colour corresponds to Annexin V‐positive, whereas green colour corresponds to Annexin V‐negative). The percentage of Annexin V stained cells is in accordance with the data obtained with c‐PARP. Androgen‐treatment cells at SAL showed a higher percentage of cell survival than control‐treated cells (Figure 3G). Notably after co‐culture, both the R1881‐ as well as the ENZ‐treated population exhibited significantly less Annexin V‐stained cells compared to control‐treated (solvent DMSO control) population. Thus, the data suggest that AR agonist and AR antagonist treated cells are resistant to lymphocyte‐mediated apoptosis.

Taken together, cell survival of each LNCaP and C4‐2 cell lines is reduced by lymphocyte co‐culture by enhancing apoptosis indicating that LNCaP and C4‐2 cells were killed by activated lymphocytes. Yet, the data also suggests that AR ligand treated PCa cells are resistant to lymphocyte‐mediated killing, where androgen‐treated PCa cells seem to be more resistant.

### 2.3 Conditioned media from AR ligand‐treated PCa cells do not affect lymphocyte‐mediated apoptosis

Immune cell attraction leading to tumour cell clearance is probably the main tumour suppressive role of SASP.^18^, ^19^ In general, immune cells can bind to SASP factors or take up exosomes being secreted in the tumour microenvironment. In addition immune cells can also bind to cell surface antigens presented by tumour cells. It is therefore suggested that the attraction and response of immune cells depends on the balance between immune cell attractant and the suppressive acting SASP factors. This led to the hypothesis that agonist and antagonist may induce a distinct secretome in PCa cells in order to regulate immune cell response. Here, we analysed the functional effect of lymphocyte‐mediated cytotoxicity using conditioned media of treated PCa cells. The conditioned media were derived from DMSO solvent treated control cells, (Con. D), R1881‐treated (Con. R) or ENZ‐treated cells (Con. E). The conditioned media were obtained from AR ligand treated PCa cells that were ligand treated for 3 days, washed and thereafter cultured for further 2 days in serum‐free media. These conditioned media contain secreted factors from SAL‐treated, Enz‐treated, or control (DMSO)‐treated cells.

**FIGURE 4:**
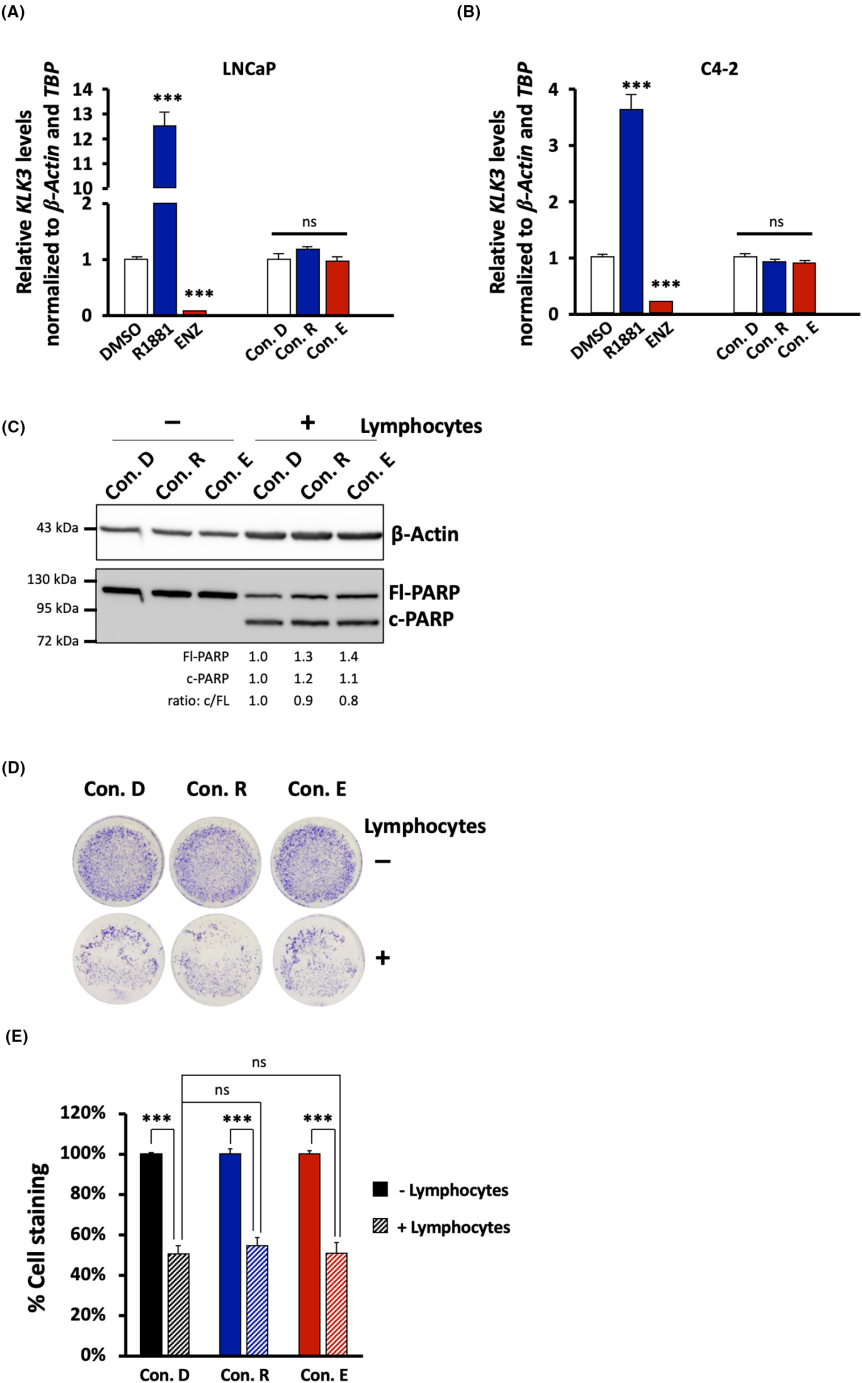
Conditioned media do not affect transcription level of AR target gene *KLK3* and lymphocyte‐mediated apoptosis. PCa cell lines were treated with AR ligand for 3 days to induce cellular senescence. Cells were washed twice with PBS and incubated further with serum free media for 2 days. Conditioned medium derived from R1881‐(Con. R), ENZ‐ (Con. E), and DMSO‐treated cells (Con. D) as control were collected. (A, B) LNCaP cells (A) or C4‐2 cells (B) were treated with indicated AR ligands or conditioned media for 6 days. RNA extraction was performed and the expression of the direct AR target gene *KLK3*, the PSA encoding gene, was analysed by qRT‐PCR. Bar graphs are shown as mean + SEM from total of six technical replicates (*n* = 6) for LNCaP and three technical replicates (*n* = 3) for C4‐2 cells. *β‐Actin* and *TBP* served as housekeeping genes. Normalized mRNA levels of control‐treated cells were set arbitrarily as 1. For AR ligand treatment, statistical analysis was performed by using two‐tailed unpaired *t*‐test compared each treatment to control treatment. (C) Conditioned media do not affect lymphocyte‐mediated apoptosis. LNCaP cells were treated for 4 days with Con. D as control, Con. R or Con. E. Thereafter, PCa cells were co‐cultured with or without activated lymphocytes in a ratio of 1:5 (PCa cells: lymphocytes). Protein extraction was performed after co‐culturing for 1.5 h. Detection of full‐length PARP (Fl‐PARP) and cleaved PARP (c‐PARP) was conducted by Western blotting and normalized to β‐Actin. Upper and middle numbers indicate normalized FL‐PARP and c‐PARP band intensities relative to Con. D + lymphocytes. Lower numbers indicate the ratios of c‐PARP versus FL‐PARP levels. (D) Representative pictures of crystal violet staining after co‐culturing for 6 h. (E) Percentage of cell survival using crystal violet staining. Cell survival without lymphocyte co‐culture was set arbitrarily as 100%. Bar graphs are shown as mean + SEM from total of four technical replicates (*n* = 4) of two independent experiments. Statistical analysis was performed by using one‐way anova followed by Bonferroni's multiple comparisons test. *** *p* ≤ 0.001; ns, not significant.

As control, to test for remaining AR ligand activity in the conditioned media, we treated LNCaP and C4‐2 cells with the conditioned media to detect changes in the expression of *KLK3*, a direct AR target gene that encoding the diagnostic marker for PCa, the prostate specific antigen (PSA). As expected, the treatment of LNCaP and C4‐2 cells led to an induction of *KLK3* expression by R1881 and repression by ENZ (Figure. 4A,B). The fold induction of *KLK3* gene expression is much lower in C4‐2 cells compared to LNCaP cells which is likely due to higher AR activity in C4‐2 cells.^16^ However, no significant change of endogenous *KLK3* expression was observed by treating cells with the indicated conditioned media in both cell lines (Figure 4A, B).

To analyse the effect of conditioned media on lymphocyte‐mediated killing, the ex vivo‐activated and clonal‐expanded lymphocytes were used for lymphocyte‐mediated killing assays of PCa cells, which was performed by co‐culturing conditioned media‐treated LNCaP cells with activated lymphocytes.

Conditioned media were used to treat LNCaP cells. No measurable difference of c‐PARP levels of LNCaP cells was observed among the treatments with Con. D‐, Con. R‐ or Con. E (Figure 4C). This suggests that conditioned media do not affect lymphocyte‐mediated apoptosis. Co‐culturing with activated lymphocytes induced c‐PARP levels suggesting induction of apoptosis and lymphocyte‐mediated killing of LNCaP cells (Figure 4C). Reduction of LNCaP cells of after co‐culturing for 6 h with lymphocytes is in accordance with detected c‐PARP levels (Figure 4D), suggest a potent reduction of cells by activated lymphocytes (Figure 4E) independent of AR ligands.

### 2.4 The secretome of AR ligand‐treated cells suppresse lymphocyte proliferation

Since the SASP may contain factors that regulate lymphocyte proliferation we hypothesized that the secretome of cells treated with AR ligand‐treated influence the lymphocyte expansion. To address this hypothesis, lymphocytes were cultured with conditioned media during activation and clonal expansion using a similar experimental setup as described in Figure 1 except that Con. D, Con. R or Con. E were employed. In addition, during clonal expansion in the presence of conditioned media, lymphocytes were also cultured with or without addition of IL‐2, ‐7 and ‐15. Note that, addition of these three cytokines is recommended during lymphocyte clonal expansion in the original protocol^15^, since these interleukins promote proliferation, survival and differentiation of lymphocytes.^20^

**FIGURE 5:**
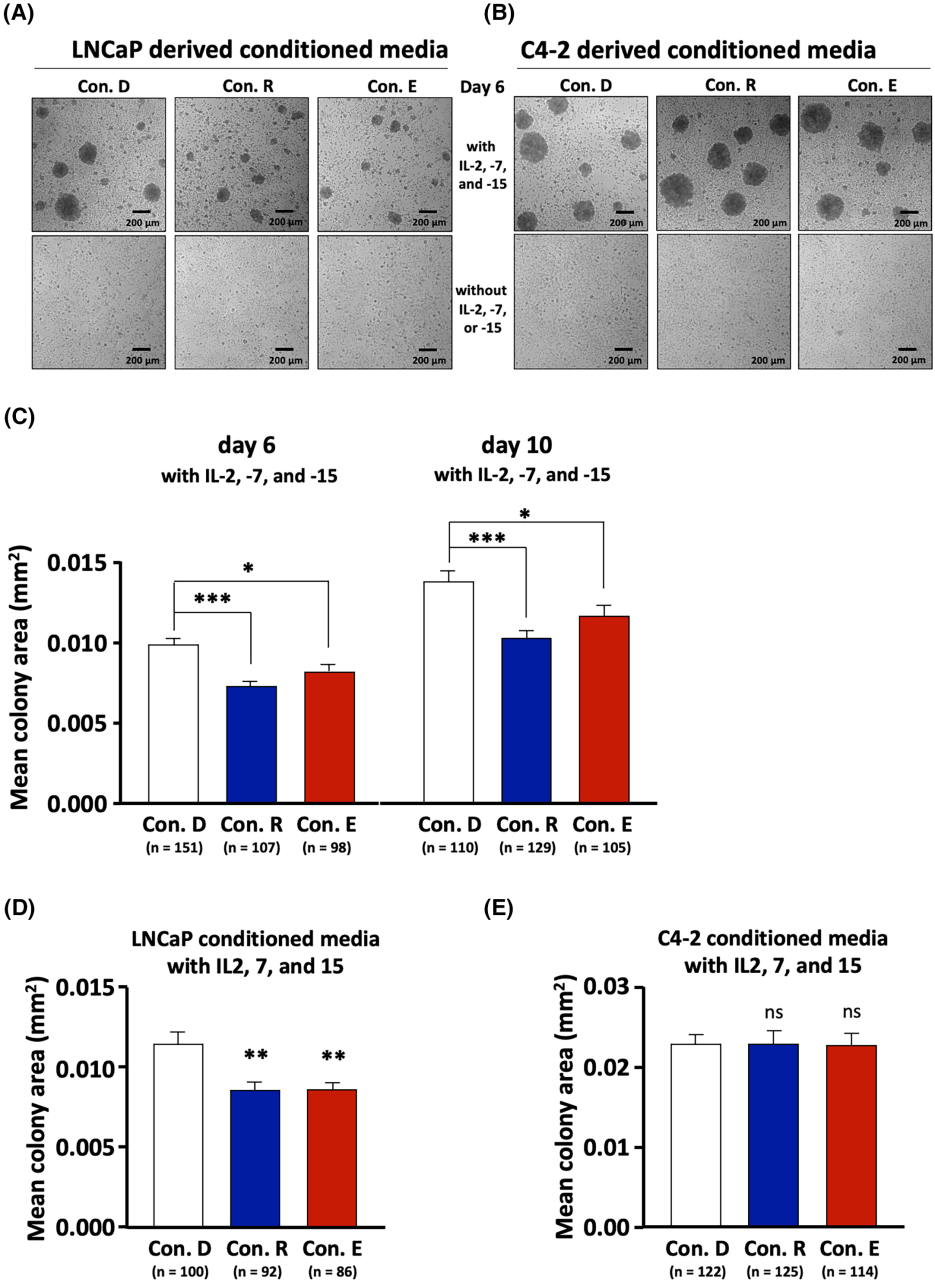
The conditioned media derived from androgen and AR antagonist treated LNCaP cells suppress lymphocyte clonal expansion. Activation and clonal expansion of lymphocytes were performed as illustrated in Figure 1 except that the conditioned media Con. D as control, Con. R and Con. E derived from LNCaP and C4‐2 cells were used. Furthermore, during clonal expansion, the activated lymphocytes were treated with or without IL‐2, ‐7, and ‐15. (A, B) Representative pictures of lymphocyte colonies after 6 days of incubation with conditioned media derived from LNCaP (A) or C4‐2 (B) with or without IL‐2, −7, and − 15 co‐treatment. (C) Mean area of lymphocyte colonies after 6 and 10 days of clonal expansion with LNCaP‐derived conditioned media with IL‐2, ‐7 and ‐15 treatment. (D, E) Clonal expansion after 6 days culturing with LNCaP (D) or C4‐2 (E) ‐derived conditioned media with IL‐2, −7, and − 15 treatment indicated as mean area of lymphocyte colonies. Measured values from conditions without IL‐2, ‐7 and ‐15 treatments were used for setting the threshold and therefore, area below 0.005 mm^2^ was considered as background (data not shown). Bar graphs show mean area + SEM calculated from colonies that exhibited an area equal to or above 0.005 mm^2^ with indicated number of colonies (*n*). Statistical analyses were performed by using one‐way anova followed by Dunnett's multiple comparisons test. ** *p* ≤ 0.01; ns, not significant.

At Day 6 of treatment differences were detected in clonal expansion comparing with and without IL‐2, ‐7 and ‐15 treatment (Figure 5A,B). As expected in the presence of the three added cytokines, colonies of lymphocytes were generated indicating a clonal expansion/proliferation. Treating with conditioned media alone, without addition of the cytokines, we did not observe colony formation. This indicates that treatment with these three cytokines are sufficient for lymphocyte clonal expansion/proliferation. Lymphocytes that underwent co‐treatment with both the cytokines and conditioned media resulted in smaller lymphocyte colonies with Con. R and Con. E compared to Con. D. This was observed after Day 6 and Day 10 of co‐treatment (Figure 5C). This suggests that conditioned medium obtained from LNCaP cells treated with R1881 or ENZ represses lymphocyte proliferation (Figure 5D). Of note, conditioned media derived from C4‐2 cells seems not to detectably affect changes in colony formation or colony size (Figure 5E). Therefore, we conclude that secreted factors of AR agonist or antagonist‐treated LNCaP cells have a stronger regulatory influence on the lymphocyte proliferation compared to the secretome derived from C4‐2 cells.

### 2.5 Co‐culture with activated lymphocytes induces cellular senescence

**FIGURE 6:**
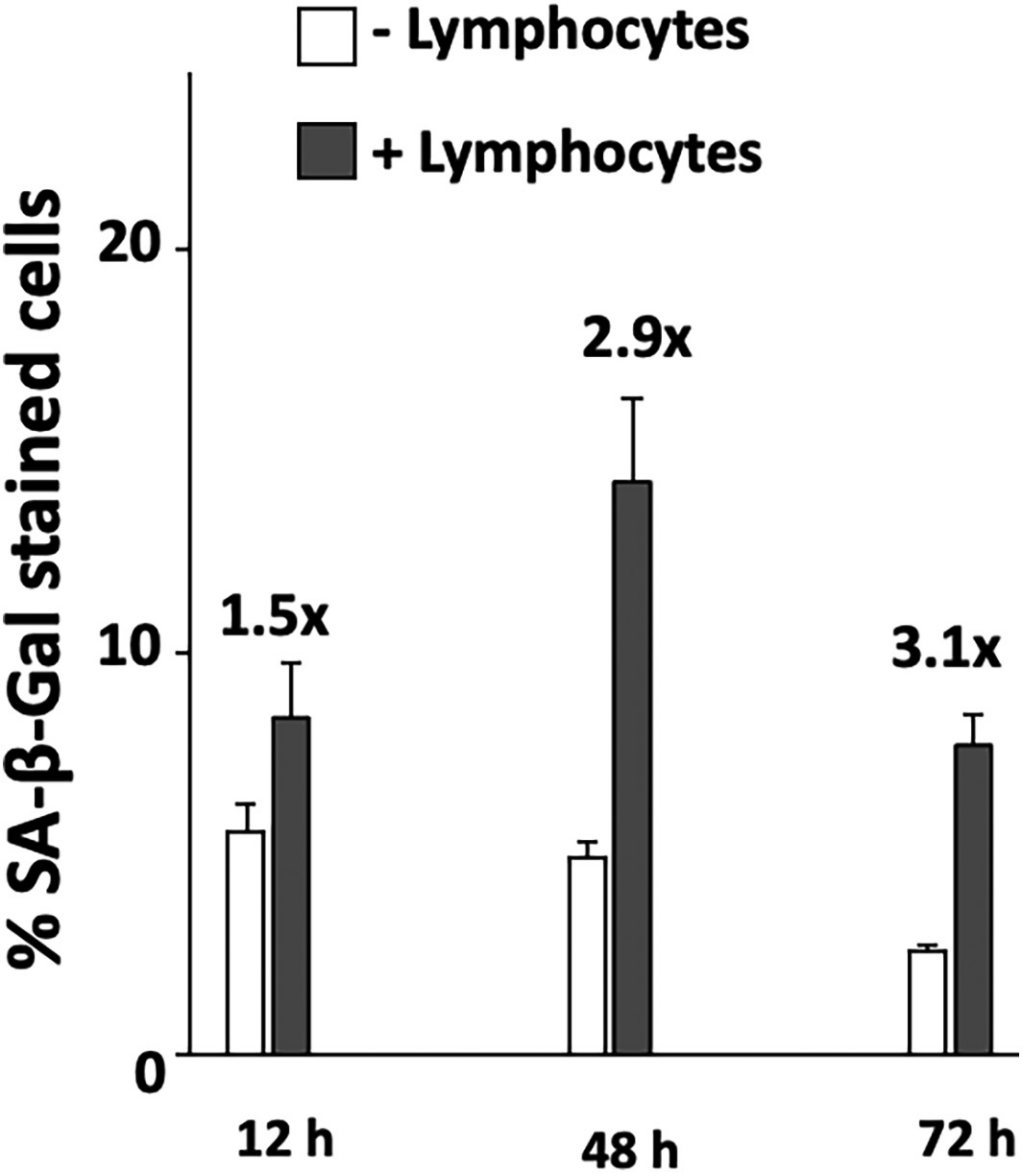
Increased senescence levels through co‐culture with activated lymphocytes. C4‐2 cells were treated with DMSO as solvent control, R1881, ENZ for either 12, 48 or 72 h. Thereafter, C4‐2 were co‐cultured with lymphocytes for 4 h in a 5:1 ratio (lymphocytes/C4‐2) prior to staining for SA‐β‐galactosidase activity. Indicated are the percentage of SA‐β‐Gal positive stained cells with error bars (SEM). The number above the bars indicate the fold increase of cellular senescence by co‐culturing with lymphocytes.

Interestingly, the accumulation of senescent cells has been suggested as a crucial factor that could tip the balance from tumour suppression to tumour promotion.^21^ To investigate a potential role of the accumulation or clearance of AR ligand‐induced senescent cells in the previously observed resistance of AR ligand‐treated cells to immunogenic apoptosis, we analysed the impact of the lymphocyte‐mediated killing, with co‐culture performed for 4 h, on senescence levels in C4‐2 cells after 12, 48 and 72 h of AR ligand treatment (Figure 6). SA‐β‐GAL staining, indicating an increase in cellular senescence levels, increased concordantly with the length of the AR ligand‐treatment period and, independent of AR‐ligand treatment, after co‐culture with activated lymphocytes. This suggests that activated lymphocytes not only induce apoptosis but also cellular senescence indicating the induction of multiple intracellular pathways.

## 3 DISCUSSION

Analysing the interplay between hormonal treatment and onco‐immunological treatments in cell culture‐based model systems could provide some relevant insights and support for onco‐immunological treatment. Many immunotherapeutic strategies have been approved for various cancer types.^22^, ^23^, ^24^, ^25^ Although several clinical trials with immunotherapeutic strategies for PCa had been started, however, since 2010, only the cell‐based vaccine Sipuleucel‐T is approved. It seems that PCa exhibits some mechanisms of immune‐suppression to limit the effectiveness of immunotherapies.^26^, ^27^

Interest in cellular senescence in the field of oncology has increased over the last years, with cellular senescence offering a potential new treatment strategy focusing on stasis rather than clearance. However, the purported tumour suppressive value of senescence through inhibition of proliferation and promotion of immune mediated tumour clearance remains highly disputed, with evidence pointing towards a tumour‐proliferative effect, likely mediated through certain types or components of the SASP.^28^

Interestingly, the accumulation of senescent cells has been suggested as a crucial factor that could tip the balance from tumour suppression to tumour promotion.^21^ The treatment of PCa cells with either AR antagonists or supraphysiological levels of androgen leads to the induction of cellular senescence in a time‐dependent manner, with a maximum observed in vitro after 72 h of treatment.^4^, ^6^ These findings suggest that AR antagonists not solely inactivate the AR and that AR antagonists themselves are neither inert nor neutralize the AR signalling. Rather antagonists induce the cellular pathway of cellular senescence in both androgen‐sensitive and CRPC as well as in native prostatectomies samples from patients.^6^ Also, it was shown that genes can be repressed by androgens as well as by AR antagonists. This indicates that AR antagonists not always antagonize the action of androgens.^5^ Thus interestingly, AR antagonists do not always exhibit the opposite activity on AR.

The paracrine effect mediated by SASP relies on the balance between secreted tumour promoting and ‐suppressive factors.^29^, ^30^ The pattern of secreted SASP factors is suggested to be cell line and treatment specific. Therefore, the composition of SASP factors within the secretome and may differ comparing cells that are exposed to agonistic or antagonistic function of AR ligands. This raises the question how AR ligands influence the tumour microenvironment as well as immune cell attraction and activity by SASP. Our data suggest an immune‐suppressive activity modulated by SASP.

In addition to findings of SASP‐suppressed lymphocyte proliferation, this study shows also that AR ligand‐treated PCa cells render PCa cells towards an immune‐tolerance phenotype since either SAL‐ or ENZ‐treated cells seem to be resistant to lymphocyte‐mediated apoptosis.

We were able to confirm the ex vivo expansion and activation of CIK lymphocytes isolated from PBMC using cytokine stimulation and activation by DCs differentiated from monocytes and show their successful use to induce immune‐mediated apoptosis in both LNCaP and C4‐2 cells. Furthermore, we were able to establish that AR ligand pretreated LNCaP and C4‐2 cells show a resistance to this immunogenic attack, with a relative reduction both of total cell loss and of apoptosis levels after AR ligand pretreatment. Moreover, while we did not observe an escape of immune attack in AR ligand‐induced senescent cells, as reported for age‐induced senescent cells, instead the cellular senescence levels are increased after immunogenic attack. This increase is, however, not observed in cells pretreated with AR‐Ligands.

Interestingly, a resistance of C4‐2 cells to immune‐cell mediated killing after pretreatment with AR‐ligands was observed. The underlying molecular mechanism is unclear.

The cell surface and downstream signalling of both lymphocytes and their target cells is required for a proper interaction in order for lymphocytes to mediate apoptosis. While some antigens on cancer cells serve as targets for lymphocyte to recognize and kill cancer cells, some surface antigens can inhibit lymphocyte‐mediated killing.^31^, ^32^ For example, cytolytic inhibitory signals can be mediated by interaction with PD‐L1 on the cancer cells with the PD‐1 receptor on cytotoxic T cells. Note that a negative correlation between AR and PD‐L1 expressions has been reported.^33^ Further, the possibility that AR ligands might change the expression of PCa cell surface proteins should be considered that could reduce or block immune cells to interact specifically with and kill PCa cells. Besides changes of cell surface factors by AR ligands, it should be included in the consideration that anti‐apoptotic signals could activated in treated PCa cells to evade immune killing.

It is known that cellular senescence protects from apoptotic pathways by activating pro‐survival pathways.^34^ In recent years it emerged that inhibiting pro‐survival pathway in senescent cells by small molecule inhibitors (senolytics) will induce apoptosis indicating that the pro‐apoptosis pathway is suppressed by the senescence pathway.

An apoptotic resistance is suggested to be one of the hallmark features of senescent cells.^35^, ^36^, ^37^ Senescent cells possess upregulated pro‐survival/anti‐apoptotic signalling pathways.^37^, ^38^ In fact, our previous findings identified the activation of the pro‐survival/anti‐apoptotic AKT signalling by androgens and Enz treatment of PCa cells.^6^, ^14^

A great amount of research will be needed to break through the barrier between the development of effective onco‐immunological treatments and their use in PCa, likely requiring a shrewd combination of multiple different treatment options to contribute to the understanding of the interactions between established hormone‐based therapies and onco‐immunological treatments in the treatment of advanced PCa.

## 4 MATERIALS AND METHODS

### 4.1 Cell culture

Preparation of PBMCs was adapted from Junking et al.^15^ Isolation of PBMCs was performed with Lymphoprep™ solution. For each experiment, 36 mL of blood sample from healthy male donor was collected in S‐Monovette® EDTA K3 tubes and further processed with Lymphoprep^TM^ solution according to the manufacturers' protocol (STEMCELL Technologies). Isolated PBMCs were incubated with serum free AIM‐V medium (Gibco, Life Technologies) in 6‐well culture plate in a 5% CO_2_ incubator at 37^o^C. After 2 h of incubation, nonadherent cells were gently collected and cryopreserved as a source of lymphocytes. After removing nonadherent cells, adherent cells were further cultured as a source of DC.

Adherent PBMCs were cultured in serum free AIM‐V medium that was supplemented with 50 ng/mL GM‐CSF and 25 ng/mL IL‐4 (both ImmunoTools) for 5 days. This leads to differentiation into immature DCs. Thereby, the medium and cytokines were daily refreshed. Differentiation into mature DCs was performed by culturing in serum free AIM‐V medium with added 50 ng/mL TNF‐α and 50 ng/mL IFN‐γ (both ImmunoTools) for further 2 days. Successful cell differentiation was confirmed by analysing cell morphology with light microscopy. These mature DCs were used for lymphocyte activation. In order to activate lymphocytes, the nonadherent PBMCs were co‐cultured with mature DCs for 3 days in AIM‐V medium with 5% human serum (Sigma‐Aldrich). The activated nonadherent PBMCs were thereafter clonally expanded in serum free AIM‐V medium supplemented with 20 ng/mL IL‐2, 10 ng/mL IL‐7 and 20 ng/mL IL‐15 (ImmunoTools) being refreshed every 2 days. Flow cytometry was used to verify successful activation and proliferation/expansion of lymphocytes.

Culturing and ligand‐treatment of LNCaP and C4‐2 cells were performed as described before.^14^, ^39^

### 4.2 Killing assays of PCa cells by immune cells

The cytolytic activity of immune cells were analysed by co‐culturing of activated lymphocytes with PCa cells at a 5:1 ratio. Cleaved PARP (c‐PARP) as apoptotic marker was determined by Western blotting as previously described^14^ with protein extraction after co‐culturing for 1.5 h. Annexin V staining with subsequent flow cytometry was used to determine the percentage of apoptotic cells after co‐culturing for 3 h. Crystal violet staining as previously described^40^ was used to detect changes of attached cell population after co‐culturing for 6 h.

Senescence‐associated β‐galactosidase (SA β‐Gal) activity staining was essentially performed as described by Dimri et al.^41^

### 4.3 Collection of conditioned media from senescent PCa cells

To analyse the effect of SASP of PCa cells, conditioned medium was collected after treatment of LNCaP or C4‐2 cells with ENZ or R1881 as AR antagonist or agonist for 72 h. Thereafter cells were washed twice with PBS to remove hormones/AR ligands and further cultured for 48 h in fresh serum‐free medium. Conditioned medium was collected and filtered through a 0.2 μm filter to remove cell debris yielding R1881‐ (Con. R), ENZ‐ (Con. E) or DMSO‐treated cells (Con. D) that were immediately used or stored at 4°C.

### 4.4 Flow cytometry of lymphocyte population

Nonactivated lymphocytes and activated lymphocytes were collected and stained with IgG FITC or IgG APC, double stained with CD3 FITC and CD8 APC antibodies or left unstained (Table 1). Cell samples were analysed for forward and sideward scatter as well as in FITC and APC fluorescent channels. Between 20,000 and 80,000 events were collected per sample, contingent on the sample concentration (events/μL), with a standard of 50,000 events. 1x PBS containing 2% FCS and 1% PFA was used to identify and exclude signals caused by solvent. The relevant lymphocyte population was gated due to cell size and density, excluding solvent and debris signals. This gate was reviewed and used for all further samples. IgG FITC and IgG APC‐stained cells were used to exclude unspecific background staining signals and establish the quadrant plot gate. The nonactivated lymphocyte population was stained for IgG FITC, IgG APC and both CD3 FITC/CD8 APC directly after PBMC extraction. The activated lymphocyte population was stained for the same markers after co‐culture with mature DCs and 8–10 days of expansion. Fluorescence levels in the corresponding wavelength channels were measured using the BD Accuri flow cytometer.

**TABLE 1:** List of antibodies used for flow cytometry.

Antibody	Company	Fluorescent marker	Dilution
IgG FITC	ImmunoTools	FITC	1:50
IgG APC	ImmunoTools	APC	1:50
CD3 FITC	ImmunoTools	FITC	1:50
CD8 APC	ImmunoTools	APC	1:50

## AUTHOR CONTRIBUTIONS

**Thanakorn Pungsrinont:** Data curation (equal); formal analysis (equal); methodology (equal); validation (equal). **Margret Ann Schneider:** Data curation (equal); formal analysis (equal); methodology (equal). **Aria Baniahmad:** Conceptualization (equal); funding acquisition (equal); resources (equal); supervision (equal); writing – review and editing (equal).

## FUNDING INFORMATION

This work was funded by DAAD to T.P. and Deutsche Krebshilfe, Germany Cancer Aid to A.B

## CONFLICT OF INTEREST STATEMENT

The authors declare that they have no competing interests

## Supporting information


Appendix S1:

## Data Availability

The data that support the findings of this study are available from the corresponding author upon reasonable request.
