# Supplementary material for: Androgen receptor agonist and antagonist reduce response of cytokine‐induced killer cells on prostate cancer cells
Source: J Cell Mol Med. 2023 Aug 28;27(19):2970–82. doi: 10.1111/jcmm.17923 (PMC10538273; doi:10.1111/jcmm.17923)
Supplement: Supplementary file 1 — Appendix S1: [file JCMM-27-2970-s001.docx]

**Supplement Fig. 1S**
